# The past is key for the future of our freshwater systems

**DOI:** 10.1038/s41467-023-39501-6

**Published:** 2023-07-06

## Abstract

Terrestrial freshwater environments are both affected by and contribute to climate change, with highly complex feedback mechanisms. We must look to the records these environments preserve of past periods of climactic upheaval to be able to prepare for an uncertain future.

Rivers and lakes have been integral to the formation and evolution of habitable conditions across the terrestrial realm throughout much of Earth’s history. They have crucial roles to play in important element cycles, weathering rocks and soils, and the transportation of key nutrients downstream. The flow of freshwater incises and floods the land, creating a plethora of diverse niches for species to occupy. Now, river systems are critical in keeping our densely populated regions habitable: supplying clean, fresh water for drinking, agriculture, and industry. How climate change will alter the flow and quality of our water supplies is a great but complex concern owing to a range of feedback mechanisms. Understanding this is crucial for preparation and mitigation; without a clean, reliable source of water, other inequalities (such as those targeted by the Sustainable Development Goals) cannot even begin to be tackled. To understand how freshwater systems may react, and contribute, to a warming climate in the future we must look to how they have responded in the past.

Here at *Nature Communications*, we have curated a Collection of high-quality research and commentary around Rivers and Lakes. This Collection highlights new approaches to studying both modern and ancient freshwater environments which have revealed and unravelled interconnections at scales from the local, to the global, and from the present day to the deep past. This shows how inseparably intertwined freshwater systems, climate, and the communities dependant on both, really are.

With freshwater systems providing the medium for many of the geological, ecological, biogeochemical, and now economic processes happening upon the Earth’s surface, it is unsurprising that they have an intricate relationship with climate. Transportation of organic matter and nutrients by rivers to the sea, or deep lakes, can facilitate carbon sequestration over geological timescales. However, freshwater systems are also sources of greenhouse gases to the atmosphere, releasing carbon dioxide and methane as carbon stocks of modern and ancient origins degrade. The relationships between freshwater systems and the climate are thus complicated, as anthropogenically driven climate warming and our environmental mismanagement alter long-balanced natural ecological and biogeochemical systems. This leads to the activation of both positive and negative climate feedback loops which may have runaway consequences.

Papers within this Collection demonstrate how, much like terrestrial carbon stocks, freshwater systems are highly susceptible to environmental change. Rapid changes in humidity and temperature are melting ice and increasing evaporative loss, altering dissolved solutes, changing acidity and nutrient availability^1^. Disruptions to regular climate patterns, increasing wildfire^2^, over management and pollution are changing regular discharge patterns and decreasing water quality.

**Figure:**
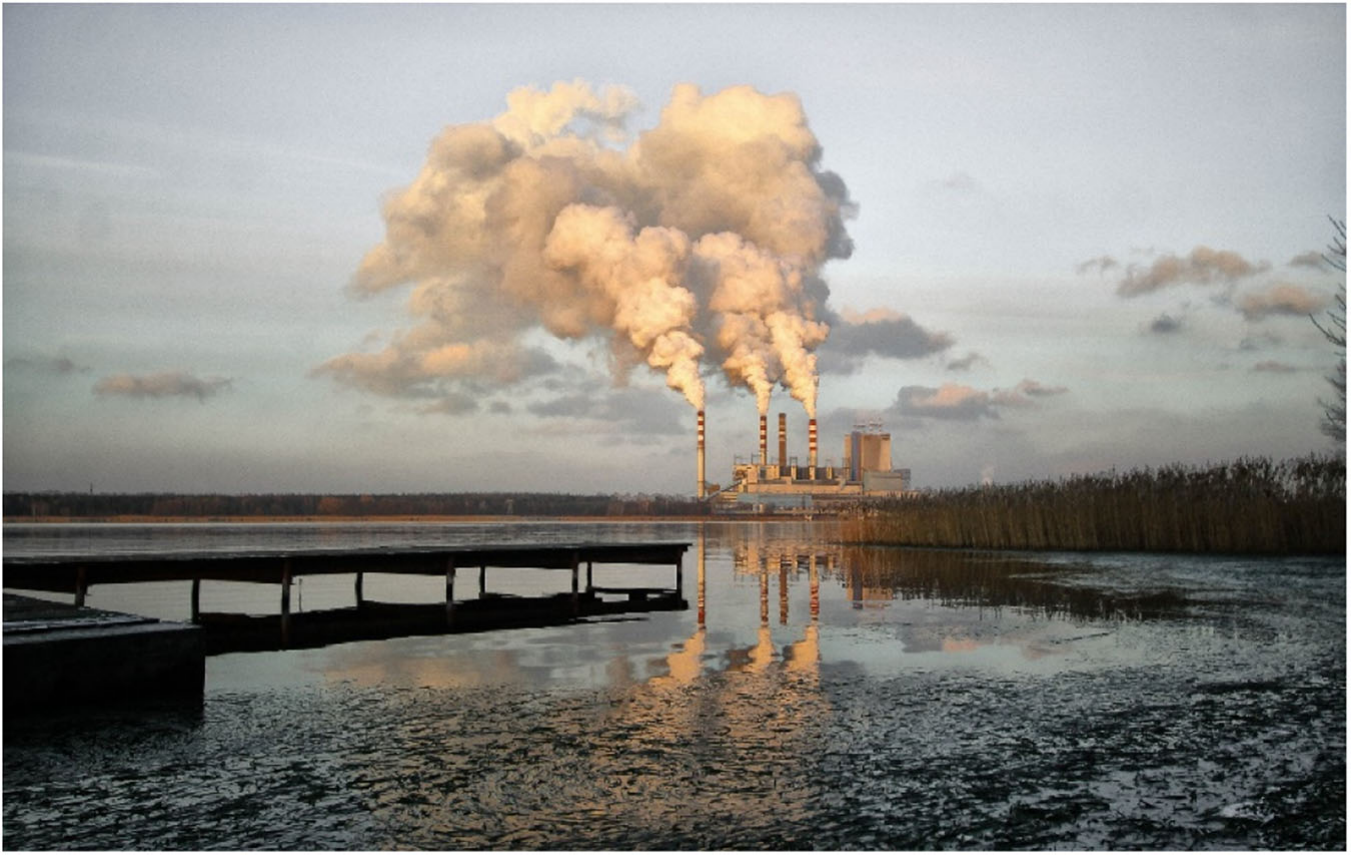
Image by Janusz Walczak from Pixabay

As well as decreasing reliability and quality of water sources for human use, these incursions on the natural states of freshwater bodies all have impacts on the ecosystems and biogeochemical cycling of elements within these systems. In particular, anthropogenic activity is leading to an increase in methane emissions from lakes due to increasing warming, eutrophication^3^, stratification^4^, and groundwater inputs^5^. As methane is a highly potent greenhouse gas, this demonstrates dangerous potential for positive climate feedbacks from freshwater systems. Worryingly, methane emissions from vast ancient lakes may have been critical positive feedback forcers in periods of past environmental turnover and crisis^6^. Despite the majority of observed feedbacks being detrimental, there are some glimmers of hope as shown by some negative ecosystem feedbacks. Warming climes are allowing beavers to expand their ranges, and biogeochemical changes brought about by their dams can temporarily offset the negative impacts increased temperatures have on water quality^7^.

Accelerated climate change will further exacerbate the detrimental changes to freshwater systems. This will all, of course, have severe effects on communities and ecosystems which rely on surface water supplies. Modelling studies show that, depending on local conditions and the predicted climate change scenario, water sources may either dry up or downstream flood risks will increase^8,9^. What is certain is that water security will decrease, yet different scenarios will require highly different mitigation strategies.

While the current pace of climate warning may be unprecedented, the key to understanding the present, and preparing for the future, is to look back to the records of the past. Deep, long-lived lacustrine systems store a wealth of knowledge in their sedimentary records. A variety of proxies may track changes in temperature, nutrient availability, oxidation state, biogeochemical processes, and ecological shifts through past periods of climate upheaval. With these palaeoenvironmental records we have the hindsight to know how these changes played out previously, especially if compared to other coeval records from distant regions. Therefore, we can disentangle short term local effects, natural variability, and unravel the wider direct controls, teleconnections and feedback processes between various more distal drivers of climate and identify triggers and warning signs of approaching tipping points.

“*While the current pace of climate warning may be unprecedented, the key to understanding the present and preparing for the future is to look back to the records of the past*.”

Lake sedimentary records detail, for example, how monsoon systems, now relied upon by billions for reliable rains, are influenced by changes at the poles. The strengths and positions of the monsoons shift with the melting and advances of ice sheets through glacial-interglacial cycles despite thousands of miles of separation^10,11^. Pollen and dust records in lake sediments show how areas have greened or desiccated in response to these changes, providing validation and nuance for predictive studies. Knowing how the hydroclimate has changed through previous periods of high CO_2_ levels and global temperatures may help both fine-tune predictions and target preparation.

Pulling together advances in all of these related fields, this Collection demonstrates how a combination of detailed monitoring in the present, predictive models looking to the future, and the complimentary study of palaeoclimate records is crucial in understanding how these vital freshwater systems will respond to climate change and how we must act now to protect them.
